# Targeted ALK inhibition achieving tumour control in metastatic melanoma: a case report

**DOI:** 10.2340/1651-226X.2026.46424

**Published:** 2026-10-01

**Authors:** Cornelia Schuster, Irja Alida Oppedal, Liv Iren Vinnem, Eirin Lunden Abrahamsson, Torill Myklestad Barrett, Eli Sihn Samdal Steinskog, Hege Elisabeth Giercksky Russnes, Åslaug Helland

**Affiliations:** a Department of Oncology, Haukeland University Hospital, Bergen, Norway; b Department of Thoracic Medicine, Haukeland University Hospital, Bergen, Norway; c Cancer Genomics, Haukeland University Hospital, Bergen, Norway; d Department of Pathology, Haukeland University Hospital, Bergen, Norway; e Department of Pathology, Division of Laboratory Medicine, Oslo University Hospital, Oslo, Norway; f Division of Cancer Medicine, Institute for Cancer Research, Oslo University Hospital, Oslo, Norway; g Institute of Clinical Medicine, University of Oslo, Oslo, Norway; h Division of Cancer Medicine, Oslo University Hospital, Oslo, Norway

**Keywords:** Cutaneous melanoma, liver metastasis, molecular targeted therapy, EML4 ALK fusion protein

## Introduction

Metastatic melanoma was historically associated with a poor prognosis because chemotherapy provided minimal overall survival (OS) and progression free survival (PFS) benefit [1]. The therapeutic landscape changed with immune checkpoint inhibitors (ICIs) and BRAF/MEK-targeted therapies, yielding substantially improved outcomes and durable long-term survival in a subset of patients [2, 3].

In Norway, PD-1 inhibition was introduced as a standard treatment for melanoma in 2015 and a combination of CTLA-4 and PD-1 inhibition in 2017. Treatment is guided by tumour burden, Eastern Cooperative Oncology Group (ECOG) performance status, serum lactate dehydrogenase (LDH), BRAF-mutation status, PD-L1 expression and American Joint Committee on Cancer (AJCC) stage (M1a–M1d). Current options include PD-1 monotherapy, PD-1/CTLA-4 and PD-1/LAG-3 combinations.

Despite 10-year OS approaching 50% after combined PD‑1/CTLA‑4 blockade, immunotherapy is frequently complicated by immune‑related adverse events (irAEs) [4, 5]; severe irAEs occur in up to 60% of patients receiving combination therapy [4].

Approximately 50% of melanomas harbour targetable BRAF V600 mutations [6]. In contrast, patients with BRAF wild-type (wt) melanoma are currently limited to ICI, although rare targetable genomic alterations, including KIT mutations and NTRK, ROS1, RET, or ALK fusions, may be identified through comprehensive molecular profiling [7, 8]. The IMPRESS-Norway trial (NCT04817956) is an investigator-initiated, national, multicentre phase II precision oncology study for patients progressing after standard therapy [9]. Since April 2021, patients have had access to comprehensive molecular tumour profiling (TruSight Oncology 500 assay, Illumina) within the public healthcare system, reviewed in weekly national molecular multidisciplinary team (MDT) meetings. Patients with actionable alterations and available matched therapy within the trial are offered treatment.

## Case report

This study describes a 66‑year‑old man with ECOG performance status 0, active and without relevant comorbidities. He was diagnosed with stage IIA melanoma on the left thorax in 2009 (pT3a, Nx). Surgical margins were negative, and no residual tumour was identified. BRAF testing was not yet implemented.

In October 2023, he developed superficial spreading melanoma of the right thigh, stage IIIB (pT2a N1c and AJCC8), BRAF-wt. Complete excision was achieved, and next generation sequencing (NGS) (Ampliseq Focus DNA, Illumina) revealed no specific mutation; RNA fusions were not assessed.

Staging Positron Emission Tomography (PET)-scan including Magnetic Resonance Imaging (MRI) of the brain (November 2023) showed suspicious liver lesions, and biopsy confirmed metastatic melanoma (M1c, AJCC8). BRAF-wt was confirmed by Quantitative Polymerase Chain Reaction (qPCR) (Thyroid Cancer Mutation Detection Kit, EntroGen), after NGS failure. Baseline imaging is shown in Figure 1.

**Figure 1:**
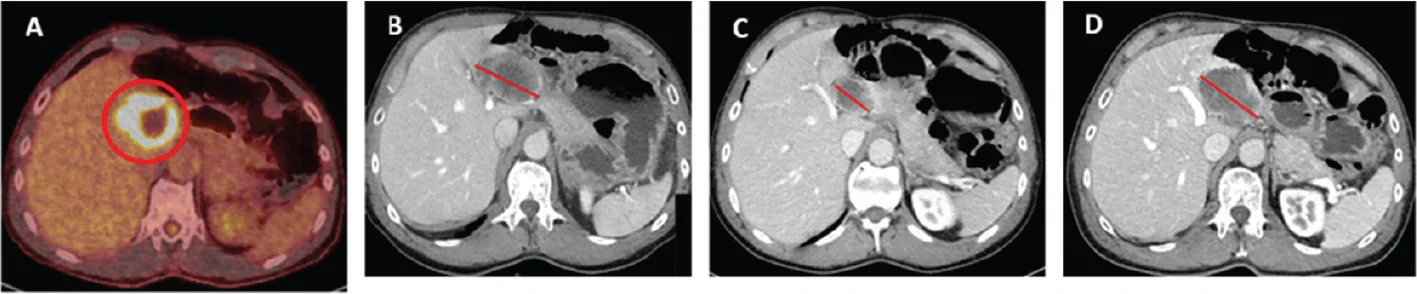
Serial radiologic response evaluations during nivolumab monotherapy. Consecutive PET-CT and CT scans obtained during treatment with nivolumab. (A) PET-CT evaluation in October 2023 and (B) diagnostic baseline CT in November 2023. (C) First response evaluation in February 2024. (D) Second response evaluation in May 2024.

Laboratory tests showed grade 1 elevations in leucocytes, C-Reactive Protein (CRP), Aspartate Aminotransferase (ASAT) and LDH.

First‑line nivolumab was started in December 2023. Both PD-1 monotherapy and PD-1/CTLA-4 combination therapy were accepted first-line options; nivolumab was chosen to minimise treatment-related toxicity. First response assessment (February 2024) demonstrated partial response (-37%, RECIST1.1 [10]), Figure 1. Treatment was well tolerated aside from grade 1 rash irAE.

In May 2024, the Computed Tomography (CT) scan revealed progressive disease (+35%), Figure 1, and the second-line ipilimumab (3 mg/kg) plus nivolumab (1 mg/kg) was initiated. At this time, both ASAT and alkaline phosphatase (ALP) were slightly increased (both grade 1), and LDH was elevated. CRP and leucocyte counts had normalised. Treatment was complicated by grade ≤ 2 diarrhoea, arthritis, fatigue, cough and reduced condition.

After two courses of treatment in July 2024, the CT scan showed further progression (53%). A repeat liver biopsy confirmed metastases with necrosis. Combined RNA and DNA analysis using the Oncomine Precision Assay (OPA, Thermo Fisher Scientific) identified an EML4(2)-ALK(20) fusion and a TP53 p.(Gly266) mutation, while KRAS, NRAS, and BRAF were wild type (wt). NRKN2-ALK and LTBP1(19)-ALK(20) fusions are not included in this panel. Given the rarity of ALK rearrangements in cutaneous melanoma, the finding was validated by fluorescence in situ hybridisation (FISH) and ALK (D5F3) immunohistochemistry, with PRAME and SOX10 positivity.

In September 2024, the patient was offered inclusion in IMPRESS-Norway. Due to the necrotic tissue, liquid biopsy (FoundationOne Liquid CDx) was performed within the IMPRESS-trial, showing high ctDNA fraction (40%), NRKN2-ALK and ALK-LTBP1 fusions, TMB 11mut/Mb, no microsatellite instability and no detectable DNA copy-number alterations. After discussion at the national molecular tumour board, the patient was offered alectinib.

Alectinib, a second-generation tyrosine kinase inhibitor binding to ALK fusion proteins, was approved in 2017 for the treatment of stage IV Non-Small Cell Lung Cancer (NSCLC) [11] and later as adjuvant treatment [12]. In our case, alectinib was initiated in November 2024 with the recommended standard dose of 600 mg twice daily (bid).

The patient discontinued treatment after 14 days due to grade ≤ 2 toxicity (myalgia, rash, constipation, peripheral oedema and symptomatic bradycardia), which impacted his quality of life. However, the CT scan in December 2024 showed partial response (-37%) compared to the baseline scan from October, Figure 2. Given the significant response and recovery from side-effects, treatment was resumed at reduced dose (450 mg bid) in January 2025 with further adjustment to 300 mg bid. Response evaluations demonstrated continued tumour regression (–46% in February and a best response of -50% in April), Figure 2.

**Figure 2:**
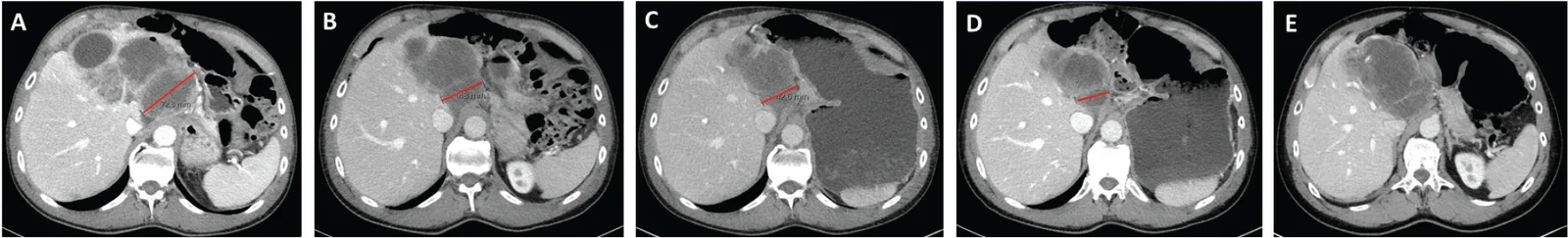
Serial CT-based response evaluations during treatment with alectinib in the IMPRESS study. Consecutive CT scans obtained during treatment with alectinib. (A) Baseline evaluation in October 2024. (B) First response evaluation in December 2024. (C) Second response evaluation in February 2025. (D) Third response evaluation in April 2025. (E) Fourth response evaluation in August 2025.

After the dose adjustments, tolerability was excellent with preserved quality of life and ECOG 0 until August 2025. The CT scan evaluation performed in mid-August showed significant progression of liver metastases (Figure 2), and the treatment was terminated.

Shortly thereafter, he was hospitalised with increasing abdominal pain, transitioned to palliative care and passed away in November 2025.

## Discussion and conclusion

The patient presented with metastatic melanoma harbouring a rare EML4-ALK fusion in the tumour tissue, whilst liquid biopsy detected distinct NRKN2-ALK and ALK-LTBP1 fusions. He experienced only short-lived benefit from PD-1 inhibition and direct progression to combination ICI, with multiple irAEs. In contrast, treatment with alectinib induced a rapid response with good tolerability after initial dose reductions. This case illustrates that rare actionable kinase fusions may identify patients deriving substantial benefit from targeted therapy beyond established BRAF-driven disease.

Although ALK fusions are established therapeutic targets in several cancers, they are uncommon in melanoma. Preclinical data suggest that sensitivity to ALK inhibition is confined to melanomas harbouring the *EML4-ALK* fusion [13]. Because of the association between ALK fusions and Spitz tumours [14], the primary tumour from 2009 was re-evaluated, confirming melanoma and the absence of ALK alterations. This case emphasises the importance of integrating histopathological assessment with comprehensive molecular profiling.

Our case highlights the complementary role of tissue and liquid biopsy in advanced melanoma, particularly when the tumour material is limited or necrotic. For clinical decision-making, the validated EML4-ALK fusion identified in tumour tissue represented the strongest evidence of an ALK-driven tumour and was considered sufficient molecular evidence to support ALK-targeted therapy. Tumour heterogeneity may result in discordant findings between tissue- and blood-based analyses. Notably, the strongly expressed EML4-ALK fusion identified in tissue was not detected in the liquid biopsy, potentially reflecting low abundance, absence or complex ALK rearrangement in circulation. Technical limitations may also contribute. Amplicon-based panels are optimised for predefined fusion transcripts and may miss rare or novel fusion partners, whereas broader cfDNA-based assays can detect atypical rearrangements but often provide limited structural resolution. Furthermore, incomplete reporting of fusion breakpoints and supporting reads may hinder independent validation and biological interpretation. These observations highlight the limitations of relying on a single testing modality. In Norway, NGS using a 50 gene panel is routinely performed for molecular characterisation in BRAF negative tumours based on immunohistochemistry. Expanded molecular profiling within Infrastructure for Precision Diagnostics (InPreD), a national precision oncology platform, can be offered to patients with ECOG performance status 0–2. Broad profiling may uncover rare actionable alterations and enable access to matched therapies through the IMPRESS-Norway trial. Since ALK inhibitors are currently only approved for NSCLC in Norway, alectinib would not have been accessible in routine clinical practice. Published evidence for ALK inhibition in melanoma remains absent. As of May 2026, no melanoma patient had been treated with an ALK inhibitor in the Dutch Drug Rediscovery Protocol (DRUP-study), NCT02925234 (personal communication). An ongoing phase II trial is investigating ensartinib in metastatic melanoma after progression to ICI and, where relevant, BRAF/MEK inhibition (NCT03420508).

Alectinib was well tolerated after early dose reduction, and the patient achieved a clinically meaningful response lasting around 9 months, with improved quality of life compared to prior ICI-therapy. Notably, a radiological response was evident at the first assessment despite only 14 days of treatment exposure. Clinical benefit was maintained despite dose reduction, consistent with evidence that alectinib dose adjustments preserve efficacy in ALK-positive NSCLC [15]. This patient achieved 9 months disease control, which compares favourably with outcomes expected from targeted therapy after ICI progression. Acquired resistance to ALK inhibition commonly arises through secondary ALK mutations or activation of bypass pathways and less frequently histologic transformation [16].

In retrospect, local treatment during maximal response may have prolonged disease control, consistent with emerging evidence supporting metastasectomy in selected patients with advanced melanoma [17].

Limitations include the single-patient nature of this report, lack of functional validation of the EML4-ALK fusion, discordant tissue and liquid biopsy findings, and the absence of repeated molecular profiling at progression to investigate resistance mechanisms.

Taken together, this case highlights the value of comprehensive molecular profiling in advanced BRAF-wt melanoma after ICI failure, as rare but actionable alterations may identify additional therapeutic options through precision oncology programs.

## Data Availability

Since this is a case report, data cannot be made available to protect the patient’s identity.
